# Rare isotope-containing diamond colour centres for fundamental symmetry tests

**DOI:** 10.1098/rsta.2023.0169

**Published:** 2024-01-22

**Authors:** Ian M. Morris, Kai Klink, Jaideep T. Singh, Jose L. Mendoza-Cortes, Shannon S. Nicley, Jonas N. Becker

**Affiliations:** ^1^ Department of Physics and Astronomy, Michigan State University, East Lansing, MI, USA; ^2^ Department of Chemical Engineering and Materials Science, Michigan State University, East Lansing, MI, USA; ^3^ Department of Electrical and Computer Engineering, Michigan State University, East Lansing, MI, USA; ^4^ Coatings and Diamond Technologies Division, Center Midwest (CMW), Fraunhofer USA Inc., 1449 Engineering Research Court,East Lansing, MI 48824, USA

**Keywords:** electric dipole moment, colour centres, diamond, symmetry violations, quantum calculations

## Abstract

Detecting a non-zero electric dipole moment in a particle would unambiguously signify physics beyond the Standard Model. A potential pathway towards this is the detection of a nuclear Schiff moment, the magnitude of which is enhanced by the presence of nuclear octupole deformation. However, due to the low production rate of isotopes featuring such ‘pear-shaped’ nuclei, capturing, detecting and manipulating them efficiently is a crucial prerequisite. Incorporating them into synthetic diamond optical crystals can produce defects with defined, molecule-like structures and isolated electronic states within the diamond band gap, increasing capture efficiency, enabling repeated probing of even a single atom and producing narrow optical linewidths. In this study, we used density functional theory to investigate the formation, structure and electronic properties of crystal defects in diamond containing ${\;}^{229}\text{Pa}$, a rare isotope that is predicted to have an exceptionally strong nuclear octupole deformation. In addition, we identified and studied stable lanthanide-containing defects with similar electronic structures as non-radioactive proxies to aid in experimental methods. Our findings hold promise for the existence of such defects and can contribute to the development of a quantum information processing-inspired toolbox of techniques for studying rare isotopes.

This article is part of the Theo Murphy meeting issue ‘Diamond for quantum applications’.

## 1. Introduction

The search for charge-conjugation-parity (CP) symmetry-violating interactions is a critical aspect of modern physics that aims to answer some of the Universe’s most fundamental questions. In particular, CP violations can help explain the observed baryon asymmetry in the Universe [1]. However, current observations of CP violations are not significant enough to account for such phenomena. Recently, the measurement of a non-zero permanent electric dipole moment (EDM) within atomic nuclei induced by the nuclear Schiff moment has garnered considerable attention as a potential solution. The existence of a permanent EDM requires breaking of both time-reversal symmetry (*T*) and parity symmetry (*P*), which, by the CPT theorem, implies that it breaks CP symmetry as well [2]. Therefore, the study of permanent EDMs in atomic nuclei provides an exciting avenue for detecting CP-violating phenomena and addressing some of the most pressing questions in physics today.

Measuring a permanent EDM poses a significant challenge due to its extremely weak signature. However, certain pear-shaped (octupole-deformed) nuclei, such as ${\;}^{223}\text{Fr}$, ${\;}^{225}\text{Ra}$ and ${\;}^{229}\text{Pa}$, have shown to be particularly sensitive to EDM measurements, making them ideal candidates for further study [3,4]. In particular, ${\;}^{229}\text{Pa}$ is predicted to provide over six orders of magnitude more sensitivity than the current experimental limit on EDM measurements taken with ${\;}^{199}\text{Hg}$ [5,6]. Despite its potential, the limited global production of ${\;}^{229}\text{Pa}$ has hindered its experimental study. However, the newly opened Facility for Rare Isotope Beams (FRIBs) at Michigan State University is expected to produce a significant amount of ${\;}^{229}\text{Pa}$ within the decade [7]. This will provide a host of opportunities to study ${\;}^{229}\text{Pa}$. One such opportunity that has been proposed is to implant ${\;}^{229}\text{Pa}$ nuclei within an optical crystal, thereby enhancing the signal for EDM measurements. This approach provides numerous advantages, such as high number densities, efficient optical probing and large internal electric fields for oriented non-inversion symmetric crystal defects in optical crystals. However, one other factor that has limited the study of ${\;}^{229}\text{Pa}$ is its extreme toxicity and radioactivity. As such, stable nuclear surrogates are necessary for the development of experimental and testing schemes prior to use of ${\;}^{229}\text{Pa}$. ${\;}^{141}\text{Pr}$ is an excellent candidate for this as it is expected to be isoelectronic with ${\;}^{229}\text{Pa}$ and has the same nuclear spin $I=\frac{5}{2}$. Moreover, ${\;}^{141}\text{Pr}$ is not as toxic and not radioactive. Thus, ${\;}^{141}\text{Pr}$ can serve as a stable nuclear surrogate to ${\;}^{229}\text{Pa}$, allowing for method development and testing. This approach can pave the way for future experiments in detecting EDMs in atoms and addressing some of the most profound questions in modern physics.

Diamond is a highly suitable host material for EDM-sensitive isotopes such as ${\;}^{229}\text{Pa}$. It possesses exceptional radiation hardness, making it more resistant to damage from implantation and the decay of incorporated radioactive species than most other host materials [8,9]. Additionally, its wide band gap (5.5 eV) increases the probability of defect formation within the gap, as demonstrated by the existence of thousands of optically active crystal defects in diamond [10]. Furthermore, synthetic diamond can be made nuclear spin-free using ${\;}^{12}\text{C}$ enriched precursors in chemical vapour deposition growth, eliminating a significant source of spin decoherence and effectively creating an almost perfect spin environment [11,12]. The extensively studied nitrogen vacancy centre in diamond can also be used as a highly sensitive quantum magnetometer and can be used for *in situ* co-magnetometry [13]. Overall, these properties make diamond a highly attractive material for hosting isotopes such as ${\;}^{229}\text{Pa}$.

This paper presents a study on the geometric structure, thermodynamic stability and electronic properties of ${\;}^{229}\text{Pa}$ and ${\;}^{141}\text{Pr}$ defects in diamond using density functional theory (DFT). Specifically, we investigate a variety of different defect configurations, including substitutional defects as well as defects with one to four vacancies introduced nearby. The paper is organized as follows: §2 outlines the computational details and methods involved in the DFT calculations; §3 presents results of these calculations, including geometric structure, formation energy, charge transition levels (CTLs), electronic structure and EDM sensitivity along with potential measurement schemes. Finally, in §4, we draw conclusions based on our findings.

## 2. Methods

Spin-polarized DFT was employed using the projector augmented wave method [14,15] as implemented in VASP 6.2.1 [16] to characterize isotopic ${\;}^{229}\text{Pa}$ and ${\;}^{141}\text{Pr}$ defects in diamond. The exchange and correlation behaviour of the valence electrons ($2s^{2}2p^{2}$, $6d^{2}7s^{2}5f^{1}$ and $4f^{3}6s^{2}$ electrons for C, Pa and Pr, respectively) during structure optimization was described using the Perdew–Burke–Ernzerhof (PBE) generalized gradient approximation [17]. To account for the strongly correlated behaviour of the *f*-electrons in actinides and lanthanides, a Hubbard-U-type correction (DFT + U) was included for Pa and Pr *f*-electrons in all PBE-level calculations. The implementation suggested by Liechtenstein *et al.* [18] was used with an on-site Coulomb parameter U=7 eV and on-site exchange parameter J=1 eV for Pa and Pr, as has been used by others to study lanthanide defects in diamond [19–22]. Additionally, the Heyd–Scuseria–Ernzerhof (HSE06) hybrid functional [23,24] was used for the calculation of highly accurate electronic structures. This range-separated hybrid functional can accurately reproduce experimental band gaps and CTLs in diamond and other group-IV semiconductors to within 0.1 eV [25,26] and has successfully described a variety of defects in diamond [26–32].

**Figure 1.**
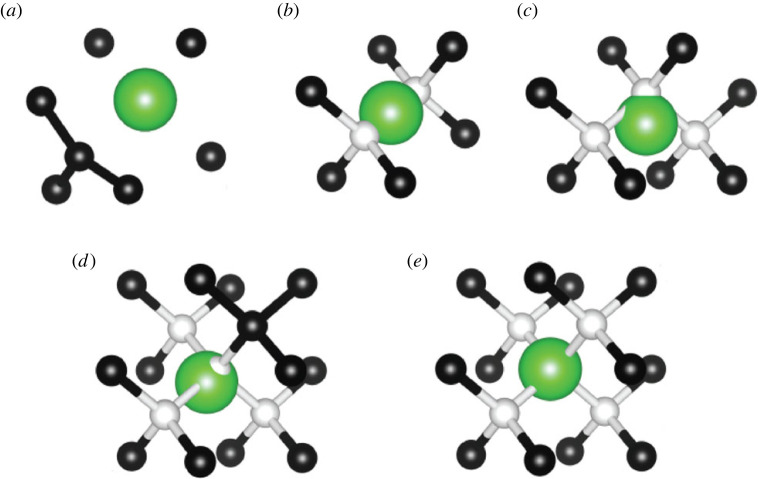
Relaxed defect structures of (*a*) ${\;}^{229}\mathrm{Pa}$${\;}_{\mathrm{sub}}$, (*b*) ${\;}^{229}\mathrm{PaV}$, (*c*) ${\;}^{229}\mathrm{PaV}$${\;}_{2}$, (*d*) ${\;}^{229}\mathrm{PaV}$${\;}_{3}$ and (*e*) ${\;}^{229}\mathrm{PaV}$${\;}_{4}$. For clarity, only the defect ion and the nearest neighbour carbon atoms are displayed. The larger green atom is the ${\;}^{229}$Pa ion. The black atoms represent carbon, while the white atoms represent vacancies. For ${\;}^{229}\mathrm{PaV}$${\;}_{2}$, ${\;}^{229}\mathrm{PaV}$${\;}_{3}$ and ${\;}^{229}\mathrm{PaV}$${\;}_{4}$ the ‘extra’ white vacancy ball that can be seen through the ${\;}^{229}\mathrm{Pa}$ is the initial position of the ${\;}^{229}\mathrm{Pa}$ at a lattice site.

A variety of defect configurations were studied, including defect ions placed in the substitutional lattice site as well as in substitutional lattice site positions with one to four vacant sites adjacent to them. Calculations were performed on a 3×3×3 diamond supercell containing 216 atoms, and the Brillouin zone was sampled at the Γ point. The excited states were calculated using the constrained-occupation DFT method (Δ-SCF) [26] with zero phonon lines (ZPL) calculated by taking the energy difference between ground and excited states. The initial geometries of the models are depicted in figure 1. The supercell defects were allowed to relax with a constant volume using a conjugate gradient method to ensure that the defect formation energies are comparable. The plane-wave energy cut-off was set to 370 eV. Ionic optimization was performed until forces were less than $10^{2}\;\mathrm{eV}\;{Å}^{-1}$, and the break condition for the electronic self-consistent loop was set to $10^{-6}\;\mathrm{eV}$ for the ZPL, hyperfine and electric field calculations. To account for the isotopic nature of ${\;}^{229}\mathrm{Pa}$ and ${\;}^{141}\mathrm{Pr}$, the mass value in the POTCAR file was changed accordingly.

The PBE functional was chosen for geometry relaxation due to its lower computational cost and its ability to predict the structures of a variety of defects in diamond with sufficient accuracy [33–36]. Furthermore, using a smaller 2×2×2 supercell of 64 atoms, we relaxed the geometry using both PBE + U and HSE06 functionals and found that the difference in atomic positions between the two relaxed structures was less than $10^{-5}\;Å$ on average, demonstrating that the accuracy of PBE + U is comparable with that of HSE06 for geometry relaxation.

To assess which defect configuration was most stable, formation and cohesive energies were calculated for each defect studied. The formation energy for a defect X with charge state q can be calculated according to:

2.1 $$E^{\text{f}}[X^{q}]=E_{\text{tot}}[X^{q}]-E_{\text{tot}}[\text{bulk}]-\sum_{i=1}^{k}n_{i}\mu_{i}+q(\epsilon_{\text{VBM}}+E_{\text{F}})+E_{\text{corr}},$$

where $E_{\text{tot}}[X^{q}]$ and $E_{\text{tot}}[\text{bulk}]$ are the total energies of the bulk material with and without the defect, respectively; $n_{i}$ is the number of atoms of species i that have been added to or removed from the supercell (for example, ${\;}^{229}{\text{Pa}}_{\text{sub}}$ removed 1 C and added 1 ${\;}^{229}\text{Pa}$); $\mu_{i}$ is the chemical potential corresponding to atomic species i; $\epsilon_{\text{VBM}}$ is the valence band maximum of the bulk material; $E_{\text{F}}$ is the Fermi level, which can have values within the material’s band gap and $E_{\text{corr}}$ is the finite-size electrostatic correction [37]. $E_{\text{corr}}$ was obtained using the scheme proposed by Freysoldt *et al.* [38] as implemented in the Spinney code package [39]. The chemical potential for C was obtained by dividing the total energy of the pristine diamond supercell by the number of atoms. The chemical potential of Pa was calculated as the total energy of metallic Pa (with a bcc tetragonal structure, I4/mmm, no. 139) divided by the number of Pa atoms. Similarly, the chemical potential of Pr was calculated using the total energy of metallic Pr (with a hexagonal structure, P63/mmc, no. 194) divided by the number of Pr atoms. For the chemical potential, the k-point sampling was increased to 9×9×9 due to the small crystal structure.

Cohesive energies were calculated according to:

2.2 $$E_{\text{c}}=\frac{1}{n}\left(\sum_{i=1}^{k}n_{i}E_{\text{atom},i}-E_{\text{tot}}\right),$$

where n is the total number of atoms, $E_{\text{tot}}$ is the total energy of the defect system, $n_{i}$ is the number of atoms of species i and $E_{\text{atom},i}$ is the energy per atom for species i [40]. In order to evaluate the cohesive energies of the structures, it was necessary to calculate the total energies of the corresponding isolated atoms in the structures (using the same exchange functionals and calculation quality settings). For the calculation of the C atom, a 10×10×10 Å cube with a single C atom in the centre was used, giving enough space around the atom for it to be considered as an isolated atom. For the Pa and Pr atoms, a slightly larger cube of 15×15×15 Å was used to ensure isolation of the atoms.

To determine the most stable charge state for a given defect, its CTLs are calculated. The CTL is the Fermi level at which a transition between two charge states becomes energetically favourable [41]. It is calculated using the formula:

2.3 $$\epsilon \left(q_{1}|q_{2}\right)=\frac{E_{q_{1}}^{\text{tot}}+E_{q_{1}}^{\text{corr}}-E_{q_{2}}^{\text{tot}}-E_{q_{2}}^{\text{corr}}}{q_{2}-q_{1}},$$

where $E_{q}^{\text{tot}}$ is the total energy of the supercell calculation in charge state q and $E_{q}^{\text{corr}}$ is the corresponding charge correction that accounts for the periodic interaction of charges between neighbouring supercells [38,42,43].

The formation and cohesive energies were evaluated for the neutral charge states of the different defect configurations to determine which configuration is most stable. From there, we limited our analysis to the most stable structure and plotted the formation energies for different charge states as a function of Fermi level to determine which charge state is most stable (determined by which charge state has the lowest formation energy for any given Fermi level). The crossing points of these formation energy lines represent CTLs, where one charge state becomes more favourable than another.

Zero-field splitting (ZFS), magnetic hyperfine and electric field gradient tensors were all calculated within VASP. For the ZFS tensor in particular, we use the method by Ivády *et al.* [44] with the PBE functional, which has been demonstrated to be sufficiently accurate [34]. For all of these calculations, a higher cut-off energy of 700 eV was used. VESTA [45] was used to visualize the defect structures in addition to the wave functions, whose plane-wave coefficients we extracted using the Python class PyVaspwfc [46]. Similarly, transition dipole moments were also calculated using PyVaspwfc.

## 3. Results and discussion

### (a) Structure and stability

First, the structure of each of the defect configurations was studied to determine which is thermodynamically most likely to form during ion implantation and subsequent annealing. All defect ions were initially placed at a substitutional lattice site with nearest neighbour C atoms removed to create vacancies. The final relaxed structures for each defect configuration are shown in figure 1. Interestingly, we find that ${\;}^{229}\text{Pa}$ and ${\;}^{141}\text{Pr}$ defects form qualitatively identical structures for all defect models considered. As such, the following descriptions and images for each defect configuration apply to both.

For the substitutional defect with no vacancies, the defect ion did not move, but the nearest neighbour carbon atoms were displaced outwards. For the single vacancy, the ion moved into the split vacancy configuration, while for the higher-order vacancy complexes, it moved into a position that filled the void created by the removed carbons. It is worth noting that while the split vacancy is inversion symmetric, the higher-order vacancy complexes are not, resulting in a permanent EDM and thus static internal electric field. While this is usually avoided for quantum information processing applications as it makes defects susceptible to environmental field fluctuations, it is in fact desirable for EDM experiments, as it increases the sensitivity of EDM measurements [6].

**Figure 2.**
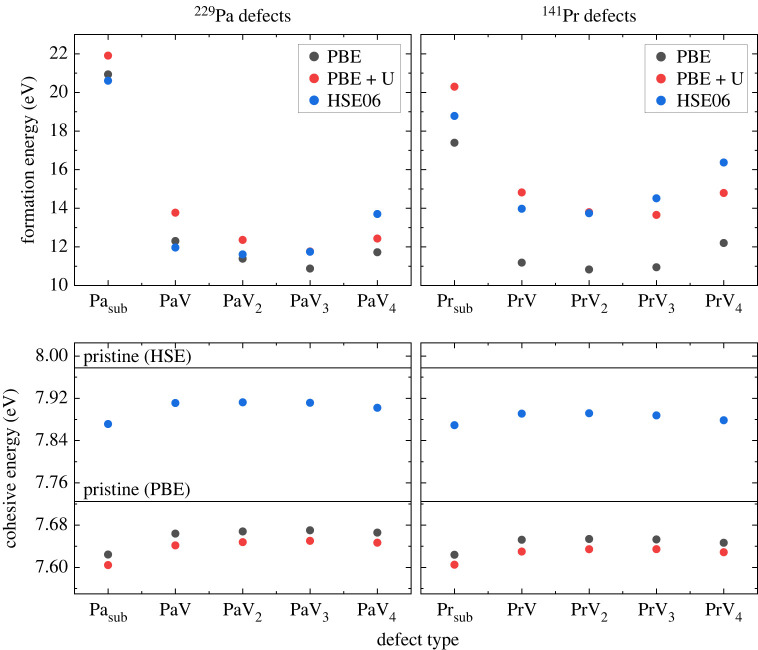
Top two panels are formation energies for different defect configurations using different functionals. Bottom two panels are cohesive energies for different defect configurations and different functionals. For the bottom panels, the solid lines denote the cohesive energy for pristine diamond without any defects using both PBE and HSE06 functionals.

The calculated formation and cohesive energies shed light on which configuration is most energetically favourable to form (figure 2). Our analysis reveals that, for both ${\;}^{229}\text{Pa}$ and ${\;}^{141}\text{Pr}$, the substitutional model is less favourable than those containing vacancies, as it generally has a higher formation energy despite having a marginally higher cohesive energy in certain cases. This is in agreement with other first principles studies of defects in diamond that feature large ions, which can introduce significant strain [20,22,36,47]. The introduction of vacancies helps to offset this by creating additional room for the dopant atom. Among the defects with vacancies, the double and triple vacancies are the most energetically favourable in terms of formation energy and have comparable or larger cohesive energy values compared with the single and quadruple vacancy defects. Between the double and triple vacancy, however, we find that the double vacancy is the most stable, as there is diminishing gains by adding yet another vacancy [19]. Moreover, higher-order vacancy complexes are kinetically less likely to form due to the low mobility of substitutional defects in diamond at typical processing temperatures [48]. Additionally, a similar *ab initio* study was done on Ce defects in diamond, and ${\mathrm{CeV}}_{2}$ was found to be most stable [20,49]. Therefore, we conclude that the most stable structure for both ${\;}^{229}\text{Pa}$ and ${\;}^{141}\text{Pr}$ defects in diamond is a defect ion accompanied by two vacancies.

**Figure 3.**
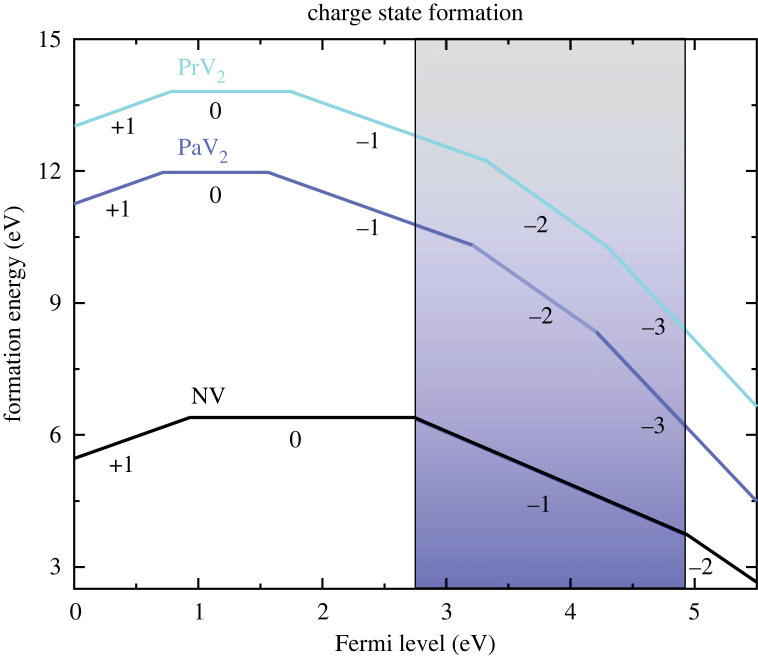
Charged formation energy as a function of Fermi level for ${\mathrm{PaV}}_{2}$ and ${\mathrm{PrV}}_{2}$ defects in diamond. Additionally, formation energies calculated by Gali [54] for the NV centre in diamond are included for comparison.

To further assess the probability of these defects forming, we compare our calculated results with values calculated for other defects in diamond. A combined experimental and theoretical study done on ${\mathrm{Er}}^{3+}$ ions implanted in diamond calculated similar cohesive and formation energies. Importantly, they also experimentally observed the characteristic telecom band emission from the Erbium ions after implantation and annealing [36]. Additionally, formation energies calculated for nickel complexes in diamond are also comparable with our values and these nickel defects are known to form in diamond [50]. Lastly, we compare the formation energies of the various charge states with the common NV centre (figure 3) and they are the same order of magnitude. These examples demonstrate the feasibility of lanthanides and actinides forming luminescent centres in diamond.

Notably, non-inversion symmetric defect configurations are preferred. As noted above, while typically not ideal for quantum information processing applications [51], the opposite is true for EDM measurements [52,53]. As was stated above, this results in a permanent electric dipole moment that can lead to linear Stark shifts. This results in symmetric doublet splittings in optical transitions when two states with oppositely oriented dipole moments are degenerate in a non-inversion symmetric site. This makes it possible to specifically address ions that have a specific direction of electric polarization. Going back to the initial statement of inversion-breaking defects being preferred, this means that for any given sample, the majority of defects formed will not be inversion symmetric and will thus retain the advantages of enhanced EDM measurement sensitivity. Based on these findings, we focused subsequent calculations on ${\;}^{229}\text{Pa}$ and ${\;}^{141}\text{Pr}$ defects with two vacancies.

### (b) Charge state formation energies

Since ${\;}^{\text{229}}{\mathrm{PaV}}_{\text{2}}$ and ${\;}^{\text{141}}{\mathrm{PrV}}_{\text{2}}$ appear to be the most thermodynamically favourable defects, which also feature promising geometries for EDM measurements, we will focus on these configurations from here on out. We start by determining their potential charge states and CTLs. Our findings indicate that both defects can potentially take on charge states ranging from −3 to +1. The formation energy as a function of Fermi level is displayed in figure 3. As mentioned above, the calculated formation energy values are comparable with those of other defects in diamond that contain large defect ions [19,36], indicating that, at least thermodynamically, defect formation is possible. Additionally, for the purposes of NV co-magnetometry, the −1, −2 and −3 charge states for both defects all land squarely within the Fermi levels where negatively charged NV centres are likely to form. Furthermore, since NV co-magnetometry requires a relatively high donor concentration, the negative charge states are preferred. In terms of which charge state is most likely to form in diamond without the need for careful doping, the −1 charge state is nearest the Fermi level for un-doped diamond [55].

CTLs were calculated using the information shown in the formation energy diagram (figure 3). Calculating the CTLs for different doping levels allowed us to determine which charge state is most stable at each Fermi level. This information is important for understanding the behaviour of the defects in diamond under different doping conditions. For example, it provides information on the effect of different atomic species on the defects’ charge stability. Here, the negative charge states act as electron acceptors and require compensatory electron donors in the system, such as substitutional N that have a deep donor level located 1.7 eV below the conduction band minimum [56,57].

### (c) Electronic structure

**Figure 4.**
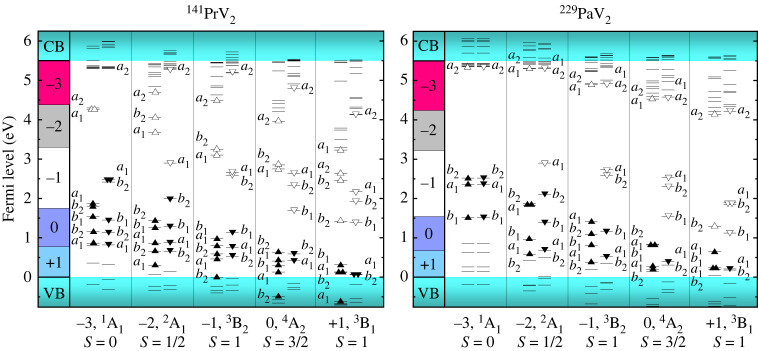
Ground state electronic structure for the charge states of ${\;}^{229}{\mathrm{PaV}}_{2}$ and ${\;}^{141}{\mathrm{PrV}}_{2}$. The single-electron orbitals are labelled with their corresponding irreducible representations.

In this section, we present a detailed analysis of the electronic structure of the defects using group theory and DFT calculations. Both defects of interest, ${\;}^{141}\text{Pr}V_{2}$ and ${\;}^{229}\text{Pa}V_{2}$, are part of the $C_{2\text{v}}$ symmetry point group. Using this, we derive a defect molecular orbital diagram to make predictions for the optical transitions and fine structure. First, we calculate the spin-polarized level structure of the single-electron orbitals using DFT and derive which irreducible representation of $C_{2\text{v}}$ they belong to by applying the respective symmetry operators to the calculated wave functions. Figure 4 shows the single-electron orbital levels obtained from the DFT calculations. From these single-electron orbitals, we construct the many electron (molecular) orbital configurations shown in figure 4. This diagram displays the single-electron Kohn–Sham energy levels and their corresponding irreducible representations, allowing possible optical transitions to be identified. From the results, the +1 and −1 charge states feature S=1 spin triplets; the neutral charge state has a S=3/2  quartet; and the −2 and −3 charge states feature an S=1/2 doublet and S=0 singlet, respectively. With this, we can identify charge states of interest based on their spin. In particular, we are interested in defect states with unpaired electrons in the ground state. This ensures that the defect is optically active and provides hyperfine coupling between a nuclear and electron spin, enabling the use of well-developed quantum control schemes to detect a nuclear Schiff moment. Specifically, nuclear spin state preparation and read out is most easily facilitated using interactions with laser light via the hyperfine coupling with a surrounding electron [58–60]. Moreover, these nuclear hyperfine states could provide potentially hour long coherence times as this has been shown in other defects with nuclear spin [61,62], further increasing the sensitivity of the measurement [63]. Given this, we limit our focus to the +1, 0, −1 and −2 charge states, leaving out the −3 charge state, which has no unpaired electrons. Importantly, it should be noted that there have been successful nuclear Schiff moment experiments that did not use hyperfine coupling [64], raising the possibility for future studies and control schemes for the −3 charge state. Among the remaining charge states of interest, we choose to further analyse the −1 and −2 charge states as they are more likely to form within natural diamond while also falling within Fermi level regions that the negatively charged NV centre does.

One additional observation from the Kohn–Sham orbitals is that the defect ions introduce occupied bands within the band gap of diamond. This differs from other colour centres in diamond such as certain group-IV or nickel vacancies where the Kohn–Sham orbitals are situated below the valence band edge [65,66]. For ${\;}^{229}{\mathrm{PaV}}_{2}$ and ${\;}^{141}{\mathrm{PrV}}_{2}$, however, both the ground and excited state levels for the minority spin channel are located within the band gap. This localization of the defect states from the bulk bands reduces the probability of single-photon transitions from the defect to bulk states during defect–defect transitions, potentially enhancing the excitation efficiency for the studied centres.

In general, our goal is to identify electronic structures featuring ground and excited state spin and orbital configurations leading to qualitatively identical interaction, splitting patterns and optical transitions for both ${\;}^{141}{\text{PrV}}_{2}$ and ${\;}^{229}{\text{PaV}}_{2}$ so that the stable ${\;}^{141}{\text{PrV}}_{2}$ defect can be used as a test bed for method development. To identify these transitions, we first need to know which optical transitions are electric dipole allowed. In $C_{2\text{v}}$, the dipole moment vector transforms as $\left({\text{B}}_{1},{\text{B}}_{2},{\text{A}}_{1}\right)$. With that, we can calculate the matrix elements for various transitions using the irreducible symmetry representation to determine which transitions are allowed. Because the electric dipole operator does not act on the spin part of the wave function, we only consider non-spin flipping excitations within the spin up and spin down channels, respectively, to identify possible optically allowed transitions. Based on ground and excited state wavefunctions, we calculate transition dipole moments to determine the strength of certain transitions. Using this, transitions which match for both ${\;}^{141}{\text{PrV}}_{2}$ and ${\;}^{229}{\text{PaV}}_{2}$ and which have a TDM>1 Debye were selected for further study. The results are displayed in table 1 along with the calculated ZPL for each transition.

**Table 1.** Matching optical transitions for the −2 and −1 charge states for ${\;}^{229}{\mathrm{PaV}}_{2}$ and ${\;}^{141}{\mathrm{PrV}}_{2}$ defects. Spin channel refers to whether a spin up or down electron was promoted to a higher band (i.e. excited state). Transition shows the symmetry of the ground and excited state. TDM is the transition dipole moment, which corresponds to the strength of the transition.

	spin channel	transition	ZPL (nm)	TDM (Debye)
${\;}^{229}{\mathrm{PaV}}_{2}^{2-}$	up	${\;}^{2}A_{1}\to {\;}^{2}B_{2}$	553	1.36
${\;}^{141}{\mathrm{PrV}}_{2}^{2-}$	up	${\;}^{2}A_{1}\to {\;}^{2}B_{2}$	731	2.88
	down	${\;}^{2}A_{1}\to {\;}^{2}B_{2}$	1763	1.05
${\;}^{229}{\mathrm{PaV}}_{2}^{1-}$	down	${\;}^{3}B_{2}\to {\;}^{3}A_{2}$	1294	6.64
${\;}^{141}{\mathrm{PrV}}_{2}^{1-}$	down	${\;}^{3}B_{2}\to {\;}^{3}A_{2}$	1378	7.71

We start our analysis with the −1 charge states for both defects, which have ${\;}^{3}{\text{B}}_{2}\to {\;}^{3}{\text{A}}_{2}$ transitions. Because both defects have $C_{2\text{v}}$ symmetry, the states that the single electron orbitals can take on can transform like $\left({\text{A}}_{1},{\text{A}}_{2},{\text{B}}_{1},{\text{B}}_{2}\right)$. These are all orbital singlets, and thus the orbital angular momentum, L, is zero for these states [67,68]. As a result, the spin–orbit coupling which is proportional to L⋅S is zero. Consequently, there is no energy splitting from spin–orbit coupling. The ground state ${\;}^{3}{\text{B}}_{2}$ features an S=1 spin triplet, and ${\;}^{229}$Pa nuclear spin I=5/2, leading to the fine and hyperfine structure shown in figure 6. Five interactions were considered when analysing the possible spin state splittings: the electron–electron magnetic dipolar interaction **D**, the hyperfine interaction **A**, the nuclear quadrupole interaction **Q** and the electronic and nuclear Zeeman interactions:

3.1 $$\hat{H}=\mu_{B}g_{e}B\cdot S+\mu_{N}g_{n}B\cdot I+S\cdot D\cdot S+S\cdot A\cdot I+I\cdot Q\cdot I,$$

where $\mu_{B}$ and $\mu_{N}$ are the Bohr and nuclear magnetons, B is the magnetic field vector, $g_{e}$ and $g_{n}$ are the electron and nuclear g-factors and S and I are the total electron and nuclear spin angular momenta.

In the principle axis coordinates, the latter three terms in equation (3.1) can be written as

3.2 $${\hat{H}}_{D}=D[S_{z}^{2}-\frac{1}{3}S(S+1)]+\frac{E}{2}(S_{+}^{2}+S_{-}^{2}),$$

3.3 $${\hat{H}}_{Q}=\frac{eQ_{I}V_{zz}}{4I(2I-1)}[3I_{z}^{2}-I(I+1)+\eta (I_{+}^{2}+I_{-}^{2})]$$

**Figure 5.**
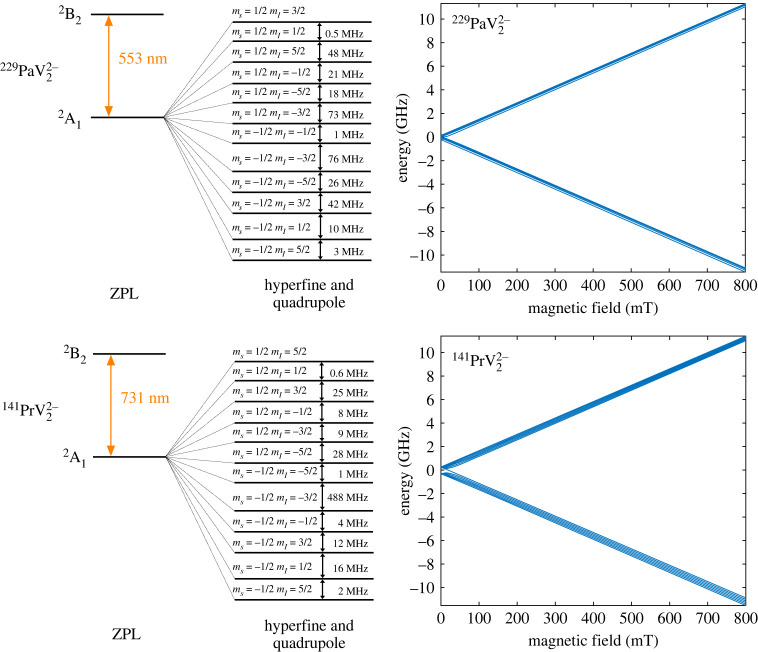
Electronic level structure with and without an applied magnetic field in the *z*-direction for the −2 charge states of ${\;}^{229}{\mathrm{PaV}}_{2}$ and ${\;}^{141}{\mathrm{PrV}}_{2}$. The magnetic field plots were simulated using EasySpin [72].

**Figure 6.**
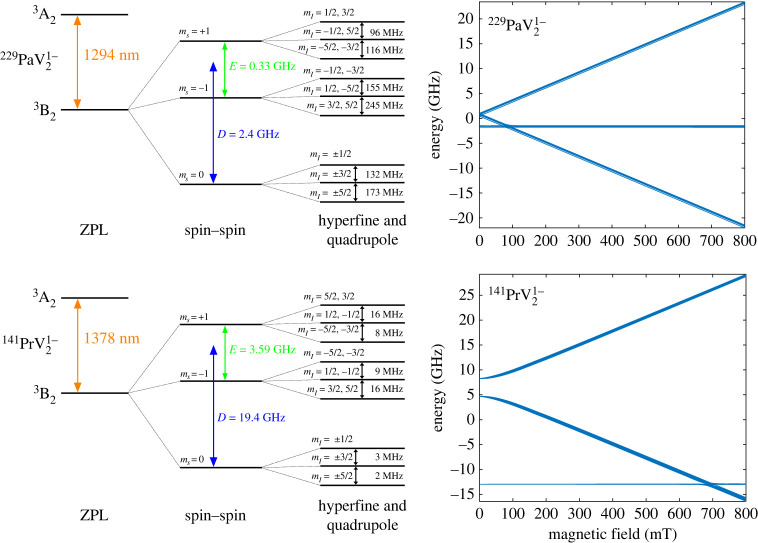
Electronic level structure with and without an applied magnetic field in the *z*-direction for the −1 charge states of ${\;}^{229}{\mathrm{PaV}}_{2}$ and ${\;}^{141}{\mathrm{PrV}}_{2}$. The magnetic field plots were simulated using EasySpin [72].

3.4 $$\mathrm{and}\;{\hat{H}}_{A}=A_{zz}S_{z}I_{z}+A_{xx}S_{x}I_{x}+A_{yy}S_{y}I_{y},$$

where $D=(3/2)D_{z}$, $E=(D_{x}-D_{y})/2$, $Q_{I}$ is the nuclear quadrupole moment, e is the electric charge and $\eta =(V_{xx}-V_{yy})/V_{zz}$ is an asymmetric coefficient [69]. It should be noted that VASP has been found to underestimate the zero-field tensor, **D**, even when using hybrid functionals, so the values may be larger than what was calculated [70]. The quadrupole for ${\;}^{229}\text{Pa}$ has not been experimentally measured, so the theoretically calculated value from Flambaum & Mansour [71] was used. The results of the DFT calculations are presented in table 2 for the defect ions of interest. With these calculated tensor parameters, we use the free software EasySpin [72] to simulate the level structure both with and without an applied magnetic field (figures 5 and 6).

**Table 2.** Symmetry labels for the ground state of each defect, hyperfine coupling parameters ($A_{xx}$, $A_{yy}$, $A_{zz}$) in MHz, electric field gradient tensor ($V_{xx}$, $V_{yy}$, $V_{zz}$) in $V\;{Å}^{-2}$, and ZFS tensor ($D_{x}$, $D_{y}$, $D_{z}$) (for S=1) in MHz for the −2 and −1 charge states. These were all calculated using the HSE06 functional except for the ZFS tensor, which used the PBE functional.

	sym.	$A_{xx}$	$A_{yy}$	$A_{zz}$	$V_{xx}$	$V_{yy}$	$V_{zz}$	$D_{x}$	$D_{y}$	$D_{z}$
${\;}^{229}{\mathrm{PaV}}_{2}^{2-}$	${\;}^{2}A_{1}$	−76.5	−71.6	89.2	422	81.2	−504	—	—	—
${\;}^{141}{\mathrm{PrV}}_{2}^{2-}$	${\;}^{2}A_{1}$	179	152	208	−199	−135	334	—	—	—
${\;}^{229}{\mathrm{PaV}}_{2}^{1-}$	${\;}^{3}B_{2}$	62.8	1.45	75.9	750	119	−870	−713	−898	1611
${\;}^{141}{\mathrm{PrV}}_{2}^{1-}$	${\;}^{3}B_{2}$	113	99.2	121	491	16.9	−507	−4696	−8255	12 951

For the level structure, we start by looking at the ZFS. The axial component of this interaction splits the $m_{s}=0$ and $m_{s}=\pm 1$ states while the rhombic anisotropy from the E term can split the $m_{s}=1$ and $m_{s}=-1$ states. The quadrupole interaction then splits all three of these branches further into $m_{I}=\pm (1/2),\pm (3/2),\pm (5/2)$ levels. From there, the hyperfine interaction splits the nuclear sublevels further. In this case, the hyperfine interaction is anisotropic, which results in more ZFS. For brevity, we have combined the splittings due to the quadrupole and hyperfine interactions in the figures. When a magnetic field is applied in the *z*-direction, the electronic and nuclear Zeeman terms split both the electronic and nuclear sublevels further as can be seen in figures 5 and 6.

The −2 charge state has one unpaired electron and therefore has spin S=1/2 due to the half-occupied molecular orbital that can be spin up or down, resulting in a spin multiplicity of 2. The ground state has the electronic configuration ${\left[a_{1}\right]}^{2}{\left[b_{2}\right]}^{2}{\left[b_{1}\right]}^{2}{\left[a_{1}\right]}^{1}{\left[b_{2}\right]}^{2}$, which transforms as the irreducible representation A${\;}_{2}$ based on the direct product of the irreducible representations that constitute the state. Similar to the −1 transitions, the excited and ground states are orbital singlets, resulting in the absence of Jahn–Teller instability and spin–orbit coupling. Similarly, there is no spin–spin interaction because there is only one unpaired electron. Thus, we start our analysis with the application of a magnetic field, which lifts the degeneracy of the $m_{s}=\pm (1/2)$ states for both the ground and excited states, as they are both spin doublets. The electronic Zeeman interaction term was given above. Similar to the −1 charge state, there are six splittings for each branch from the nuclear Zeeman and magnetic hyperfine interaction terms, which split the $m_{I}=\pm (1/2),\pm (3/2),\pm (5/2)$ levels. See figure 5 for the full level structure along with calculated values.

### (d) Potential measurement scheme

Here, we attempt to provide a general description of how a measurement using this defect could be carried out. In a typical nuclear Schiff moment search using diamagnetic atoms, a small bias magnetic field is applied to provide a quantization axis for the nuclear spin states. The magnitude of the magnetic field is chosen to be large enough to overwhelm residual magnetic fields but small enough to be generated by a low current source that can be made very stable in time. Once polarized, the nuclear spins can be readily oriented perpendicular to the magnetic field causing them to precess. This precession frequency is straightforward to measure and provides direct access to the energy difference between two nuclear spin states. If there were a non-zero nuclear Schiff moment, then this would induce an atomic EDM that would couple to an external electric field causing a shift in both the energy levels and the spin precession frequency. The shift due to the EDM can then be isolated by flipping the direction of the electric field relative to the magnetic field from parallel to antiparallel. This measurement protocol crucially relies on the magnetic field being very uniform and very stable to minimize systematic effects.

For the case of isotopes embedded in optical crystals, in this case diamond, the quantization axis is provided by the internal structure and symmetry of the defect itself. The energy difference between the two nuclear spin states can be measured using RF spectroscopy. The electric field ‘reversal’ in this case is realized by studying two sub-ensembles of defects with dipole moments oriented along opposite crystal directions (e.g. [111] and $\left[\bar{1}\bar{1}\bar{1}\right]$). The general idea is to measure the energy difference between two nuclear spin states for the two sub-ensembles separately and then to subtract them to isolate the component due to the EDM. More rigorous discussions of schemes for how this measurement protocol could be carried out in similar systems have been articulated in [6,63]. It should also be noted that preferential alignment of groups of defects has been demonstrated for NV centres in diamond and could potentially be applied here [73].

### (e) Estimated EDM sensitivity

To provide a first estimate of the sensitivity of these defects for nuclear Schiff moment and EDM measurements, we focus on the effective internal field generated by their non-inversion symmetric structure. To do this, we first attempt to estimate the differential dipole moment, Δμ, and polarizability, Δα, between the ground and excited states. We take an approach proposed by Ramachandran & Vutha [63] and Bathen *et al.* [74] where the Stark shift of the ZPL can be modelled by

3.5 $$\Delta E_{\text{ZPL}}=-\frac{1}{\epsilon_{s}}\Delta \mu E-\frac{1}{2\epsilon_{s}^{2}}\Delta \alpha E^{2},$$

where E is an applied electric field perturbing the defect, and $\epsilon_{s}$ is the static dielectric constant of the material, which we take as 5.7 for diamond [75]. Using this, we calculate the ZPL at varying applied electric field strengths and fit the equation above to the resulting ZPL energy changes. We performed these calculations for both the −1 and −2 charge state for the ${\;}^{229}{\mathrm{PaV}}_{2}$ defects. The VASP applied electric field is in units of ${\mathrm{eV}\;Å}^{-1}$, so the resulting Δμ is initially in units of e Å and Δα is in units of ${\text{m}}^{2}\text{e}\;V^{-1}$. We also provide these values in the other typically quoted units of Debye for differential dipole moment and ${\text{a}}_{0}^{3}$ for differential polarizability (table 3).

**Table 3.** Differential dipole moment and polarizabilities in two different unit systems (those from VASP, and those typically quoted). Additionally, values for the effective electric field that an electron experiences as well as the shielded value that a nucleus feels.

	Δμ (e Å)	Δμ (D)	$\Delta \alpha \;({\text{Å}}^{2}e\;V^{-1})$	$\Delta \alpha \;(a_{0}^{3})$	$E_{\mathrm{eff}}^{\mathrm{electron}}\;({\mathrm{MV}\;\mathrm{cm}}^{-1})$	$E_{\mathrm{eff}}^{\mathrm{nucleus}}\;({\mathrm{MV}\;\mathrm{cm}}^{-1})$
${\;}^{229}{\mathrm{PaV}}_{2}^{2-}$	0.065	0.313	1.536	148.47	94	0.94
${\;}^{229}{\mathrm{PaV}}_{2}^{1-}$	0.105	0.503	0.458	44.27	151	1.51

With these values in hand, we can make an estimate for the internal effective electric field within the crystal. To do this, we use the following equation:

3.6 $$E_{\text{eff}}^{\text{electron}}=\left(\frac{1}{4\pi \epsilon_{0}}\right)\left(\frac{\Delta \mu }{Z_{\text{scale}}^{3}}\right),$$

where $\epsilon_{0}=8.85\times 10^{-12}\;({F\;m}^{-1})$ is the permittivity of free space, and $Z_{\mathrm{scale}}=1\;Å$ is a length scale estimate for the volume. Importantly, the dipole moments used in the equation for differential dipole moment are not the EDM that is measured for fundamental symmetry breaking. It is the overall induced dipole because of the effective electric field within the non-inversion symmetric defect. With this, we can obtain a rough estimate for the effective electric field experienced by an electron within the defect. This estimate for the effective electric field is for the electrons, but for nuclear Schiff moment experiments, the relevant quantity is the effective electric field for the nucleus. In order to translate the effective electric field from the electrons to the nucleus, we referred to typical calculations for the effective internal electric field in polar diatomic molecules. In molecules of this type, the effective electric field for electron EDM is $10\;{\mathrm{GV}\;\mathrm{cm}}^{-1}$ while the effective electric field for the nuclear Schiff moment is $0.1\;{\mathrm{GV}\;\mathrm{cm}}^{-1}$. One example of these calculations are for RaF [76]. We apply this heuristic for the calculations presented for Pa ions in diamonds by dividing the value we found for electrons by 100 to get an estimate for the nucleus. Lastly, it should be noted that the method used to calculate the effective electric field tends to overestimate the effective electric field strength by a factor of 10 [74].

Outside of these values for an effective field and differential polarizability, the defects have several other advantages. One such advantage is that the angular momentum is zero, so this greatly reduces their coupling to the lattice, enabling narrow optical linewidths. Furthermore, in the case of the −1 charge state, it features a spin-1 triplet, which should extend its coherence time because there will be limited coupling to the spin-1/2 bath within diamond, similar to the NV centre.

## 4. Conclusion

We have identified the ${\;}^{229}{\text{PaV}}_{2}$ defect in diamond as a promising candidate for tests of fundamental symmetry violations. It lacks inversion symmetry, which allows for heightened EDM sensitivity and can also inhabit a number of negatively charged states, which have similar Fermi levels to the NV centre, enabling co-magnetometry with NV centres. Multiple optical transitions which can be captured with laser spectroscopy techniques were identified. Furthermore, a large effective electric field was calculated. Moreover, while production of ${\;}^{229}\text{Pa}$ will occur at the FRIBs, we have also identified a stable lanthanide-containing defect in the form of ${\;}^{141}{\text{PrV}}_{2}$ defects in diamond, for which we have identified ground to excited state configurations and transitions that are qualitatively identical to those of the ${\;}^{229}{\text{PaV}}_{2}$. This will facilitate experimental method development. While not considered here, the effect of applied electric fields or strain could also serve to enhance the dipole moment and the Hamiltonian terms should be explored in the future. Overall, this work establishes the concept of using rare isotopes embedded within diamond for tests of fundamental symmetries. Additionally, it highlights the potential of applying well-developed quantum control schemes to the search for a nuclear Schiff moment. This approach may also be applicable to other more stable isotopes which may also have octupole deformations [77,78] in other types of optical crystals [63].^1^

## Data Availability

The data are available in https://github.com/imorr342/IsotopeDefects.git.

## Footnotes

1 The authors recently became aware of a similar proposal in [63].
